# Radiographic and anatomical morphometric assessments of heart size in presumed healthy pet guinea pigs

**DOI:** 10.1111/vru.13020

**Published:** 2021-09-16

**Authors:** Margherita De Silva, Pierfrancesco Bo, Elisabetta Dora Genocchi, Claudio Tagliavia, Mariana Roccaro, Annamaria Grandis, Marco Baron Toaldo

**Affiliations:** ^1^ Department of Veterinary Medical Sciences University of Bologna Ozzano dell'Emilia Bologna Italy; ^2^ Veterinary Clinic Dr. Bo and Genocchi Medicina Bologna Italy; ^3^ Department for Small Animals, Division of Cardiology, Clinic for Small Animal Internal Medicine, Vetsuisse Faculty University of Zurich Zurich Switzerland

**Keywords:** anatomy, cardiology, cavia porcellus, exotics, radiography, VHS

## Abstract

Cardiac disease in guinea pigs has been reported in the literature; however, reference intervals for normal radiographic heart size obtained using objective measurement methods have not been provided for this species. The aim of this prospective, reference interval study was to describe cardiac dimensions in presumed healthy guinea pigs using the vertebral heart scale (VHS) from thoracic radiographs, as described for dogs and cats. Furthermore, an anatomical study was carried out to compare the radiographic and anatomical findings. Thoracic radiographs were acquired in right lateral recumbency for 30, client‐owned, conscious, presumed healthy guinea pigs and radiographs were acquired in left lateral recumbency for 10 presumed healthy guinea pigs as comparisons. The influence of sex, age, body weight (BW), and recumbency on the VHS and absolute cardiac measurements was investigated. The median (interquartile range; IQR) VHS was 7.4 (7.1‐7.6). No differences emerged between the VHS measured in right versus left lateral recumbency (*P* = .41) or between sexes (*P* = .16). The VHS values were not influenced by age (*P* = .53) or BW (*P* = .26). The anatomical study was carried out on 10 guinea pig cadavers, and in situ and ex situ cardiac measurements were taken using a caliper. A median (IQR) 7.5 (7.2‐8.0) VHS was assessed by this anatomical study. The reference intervals provided should be useful tools in the future for the radiographic interpretation of cardiac size in guinea pigs in clinical practice.

**Abbreviations**
Ao: aortic root
BW: body weight
CI: confidence interval
CVC: caudal vena cava
ICC: intra‐class correlation coefficient
IQR: interquartile range
IS: intercostal space
IVS: interventricular septum
LA: cardiac long axis
LAD: left atrium diameter
LVID: left ventricular internal diameter
LVPW: left ventricular posterior wall
RVID: right ventricular internal diameter
SA: cardiac short axis
T4: fourth thoracic vertebra
T5: fifth thoracic vertebra
v: units of vertebral length
VHS: vertebral heart scale.

## 1 INTRODUCTION

Cases ofpet guinea pigs (*Cavia porcellus*) presented having signs indicative of cardiac disease in clinical practice have been reported in the literature.^1^, ^2^, ^3^, ^4^, ^5^ Common clinical signs of cardiac disease are non‐specific, such as dyspnea, lethargy, and anorexia.^6^ It has been shown that only a minor percentage of guinea pigs with cardiac disease show heart murmurs on auscultation and that, on echocardiography, dilated cardiomyopathy is most frequently diagnosed in association with secondary pericardial effusion.^5^ Instead, hypertrophic cardiomyopathy and valvular disease, in association with pleural effusion, are evidenced in a minority of cases.^5^ Cardiomegaly, pleural effusion, and pulmonary edema are often present on thoracic radiographs in cardiopathic patients.^6^

Standard thoracic radiography is the diagnostic method of choice in small exotic mammals for the assessment of the trachea, the lung parenchyma, and the pulmonary vessels. However, it is also routinely utilized for a first evaluation of the heart and the large blood vessels as well as for monitoring heart size and cardiac chamber changes over time and/or in response to treatment,^7^ together with the utilization of more advanced and specific diagnostic techniques, such as ultrasonography, which should be performed in a complete cardiological assessment.^8^ The guinea pig thorax is short, and the precordial section is only one‐to‐three intercostal spaces wide.^8^ The heart occupies a relatively large space in the thorax, and it lies along the midline from the second to the fourth intercostal space.^8^, ^9^

The vertebral heart scale (VHS) system, as described for dogs by Buchanan and Bucheler,^7^ is an objective standardized diagnostic technique that is well established for dogs of various breeds^7^, ^10^, ^11^, ^12^ and cats,^13^ but is being increasingly utilized in exotic companion mammals as well. To date, it has been used for the cardiac assessments of rabbits,^14^, ^15^ chinchillas,^16^ ferrets,^17^ mice,^18^ and African hedgehogs,^19^ among others. The accuracy of thoracic radiography in detecting deviations in the cardiac silhouette relies on the availability of species‐specific normal reference data. In guinea pigs, however, the reference intervals for normal heart size derived from objective radiographic measurement methods have not yet been provided, causing clinicians to subjectively assess the presence of abnormal changes in the cardiac silhouette in clinical practice; for instance, associated signs, such as tracheal elevation, are currently being used as an aid for determining the presence of cardiac enlargement from thoracic radiographs.^6^ The availability of species‐specific reference ranges of normality regarding heart size could allow helping with the objective radiographic interpretation of the cardiac silhouette.

The aims of the present study were: (a) to assess the feasibility of radiography in evaluating cardiac dimensions in conscious, presumed healthy pet guinea pigs; (b) to establish reference intervals of the normal cardiac dimensions in guinea pigs using the VHS from thoracic radiographs; (c) to compare the cardiac measurements obtained from anatomical specimens with radiographic findings as well as with the echocardiographic values reported in the literature; and (d) to assess the influence of age, sex, body weight (BW), and recumbency on cardiac size.

## 2 MATERIALS AND METHODS

### 2.1 Radiographic study

In this prospective, reference interval study, 30 client‐owned pet guinea pigs, 14 males and 16 females, 2.4 ± 1.5 years of age, and weighing 972 ± 193 g, were brought to a private veterinary clinic for the purpose of health consultation between February 2019 and July 2019. The hospital director approved the use of the data. Included guinea pigs were presumed to be healthy if they had no history or clinical evidence of cardiovascular or pulmonary disease (such as asthenia, coughing, dyspnea, abnormal mucous membrane coloration on physical examination, or heart murmurs, extra heart sounds, arrhythmias, or abnormal lung sounds on auscultation). All the guinea pigs underwent a full clinical examination and cardiac auscultation carried out by a veterinarian with expertise in exotic animal medicine (PB).

Two additional client‐owned guinea pigs (3 and 5 years of age) that had been diagnosed with cardiac disease were also radiographically evaluated in the study, purely for a preliminary comparison, to show VHS changes in cardiomegaly. These guinea pigs presented with lethargy, dyspnea, and a heart murmur on auscultation and were both echocardiographically diagnosed with a dilated cardiomyopathy and left‐sided heart insufficiency, with eccentric left ventricular hypertrophy.

All the radiographic examinations were performed using a digital tabletop radiographic unit (UNIVET 300 HF, Multimage s.r.l., Cavaria, Varese, Italy) and digital storage phosphor plates (Agfa CR MD1.0 General Set; Agfa HealthCare, Morstel, Belgium), utilizing 44–60 kVp and 4–5 mAs settings. The guinea pigs were unsedated and manually restrained on the radiographic table in right and left lateral recumbency with the pectoral limbs pulled forward by one veterinarian trained in exotic medicine (PB) and one trained assistant (MDS). X‐ray foam positioning blocks were used to ensure easier patient positioning and to reduce the stress associated with physical handling. In addition, the primary beam was adequately collimated to the thorax only. Thoracic radiographs in right lateral recumbency were obtained from all 30 guinea pigs, while the radiographs in left lateral recumbency were obtained from only 10 (of the 30) guinea pigs as a comparison and to assess the influence of recumbency on VHS measurements.

Radiographs were scanned (EXAMION® X‐CR Smart Pure, Fellbach, Germany), acquired on a PC workstation (ThinkStation P320, Lenovo Group Ltd., Beijing, China), stored, and later analyzed using an image analysis workstation (EXAMION® AQS VET Software 3.2.12.0, Fellbach, Germany).

For each variable, the value recorded represented the mean value of the measurements taken by three different veterinarians having a medium (M.D.S.) or high level of clinical expertise (E.D.G., P.B.) in exotic small mammal practice. Each observer was blinded to the results of the measurements taken by the other two observers. The cranio‐caudal extension of the heart, as well as the number of intercostal spaces occupied by the cardiac silhouette on the radiographs, were recorded and analyzed.

The following measurements were obtained from the right and left lateral projections:

Cardiac long axis (LA) from the ventral border of the left mainstem bronchus to the cardiac apex;

Cardiac short axis (SA) taken at the middle third of the heart at its widest point, perpendicularly to the long axis. The two measurements were transposed over thoracic vertebral bodies (starting from the cranial edge of T4) and expressed as units of vertebral length to the nearest 0.1 vertebra (v);

Sum of the cardiac length (LA) and width (SA) measured in millimeters;

VHS in units of vertebral length (sum of the LA and the SA in vertebrae, as previously described for the dog by Buchanan and Bucheler)^7^;

Maximal diameter of the caudal vena cava (CVC) and

Length of the vertebra dorsal to the tracheal bifurcation (T5).

**FIGURE 1:**
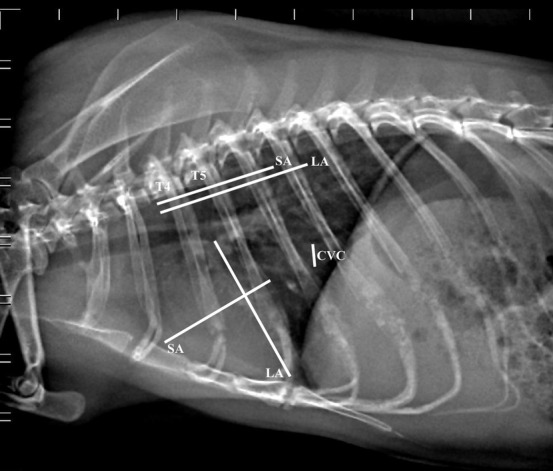
Right lateral thoracic radiograph of a clinically normal guinea pig illustrating measurements for the evaluation of cardiac size (60 kV, 200 mA, 20 mS, 4 mAs). CVC: maximal width of the caudal vena cava; LA: apicobasilar length of the heart; SA: width of the heart at its widest point perpendicular to LA; T4: fourth thoracic vertebra. T5: length of the fifth thoracic vertebral body; The LA and SA dimensions have been transposed onto the vertebral column starting from the cranial edge of the body of T4, calculated in units of vertebral length, and added to yield the VHS

The measurement technique from the right lateral view is illustrated in Figure 1.

### 2.2 Anatomical study

An anatomical investigation of the guinea pig heart was carried out for comparison with the radiographic findings acquired in the present study and with the echocardiographic measurements reported in a previously published study.^20^ For this purpose, 10 guinea pigs, three females and seven males, weighing 544 ± 75 g, that had died of diseases other than those affecting the cardiovascular or pulmonary systems, were utilized. According to Directive 2010/63/EU of the European Parliament and of the 22 September 2010 Council regarding the protection of animals used for scientific purposes, Italian legislation (D. Lgs. n. 26/2014) does not require any approval by competent authorities or ethical committees as the anatomical investigation in the present study did not influence any therapeutic decisions.

Each fresh body was kept at refrigerator temperature (+4°C) for no more than 5 h. The heart was examined by anatomical dissection in situ and ex situ. The topography of the heart was investigated after placing the guinea pigs in right lateral recumbency, removing the left lateral thoracic wall, resecting the left front limb, and removing the left ribs from the second to the ninth. All the anatomical measurements were recorded by two veterinarians (M.D.S., A.G.), each blinded to the results obtained by the other, and the measurements were then averaged.

**FIGURE 2:**
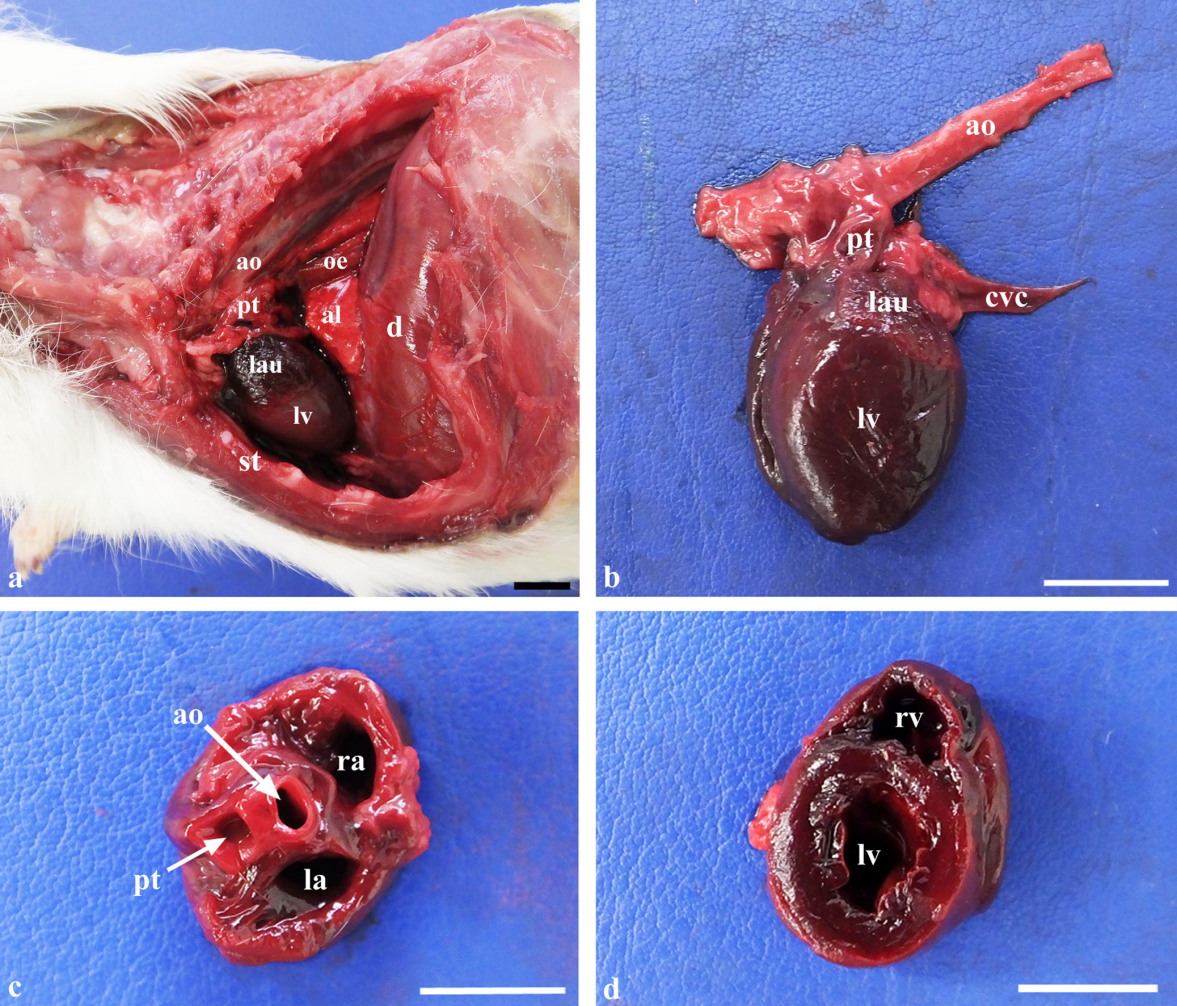
Photographs illustrating anatomical specimens of the guinea pig heart in situ and ex situ. **A,** *In‐situ* topography of the heart with the animal positioned in right lateral recumbency after resection of the left ribs from the second to the ninth and excision of the left lung. I, first rib; al, right accessory lung lobe; ao, aorta; d, diaphragm; lau, left auricle; lv, left ventricle; oe, oesophagus; pt, pulmonary trunk; st, sternum. **B**, Ex situ left lateral view of the heart. cvc, caudal vena cava. **C,** Dorsal view of the heart cut transversely at the level of the cardiac base, showing the atria and the aortic and pulmonary annulus. la, left atrium; ra, right atrium. **D,** Dorsal view of the heart cut transversely at the midventricular level. lv, left ventricle; rv, right ventricle. Scale bar: 1 cm [Colour figure can be viewed at wileyonlinelibrary.com]

The cranio‐caudal extension of the heart in situ was recorded. Cardiac long and short axis measurements were taken in situ using an electronic digital caliper (Kennon instruments^®^; 0 to 15 cm measuring range; 0.01 mm increments for precise measurements, accuracy within ± 0.03 mm), and were then compared with units of vertebral length starting from the cranial edge of the body of T4, expressed both as units of vertebral length and as absolute measurements (mm), as in the radiographic technique as shown in Figure 1. After ligation of the great vessels close to the heart, and excision of the heart at the cardiac base, the heart was cut transversely at the level of the aortic annulus and atria, and at the level of the papillary muscles of the left ventricle to expose the cardiac chambers, attempting to reproduce the standard echocardiographic views (Figure 2).^21^ At the aortic root level, the internal diameter of the left atrium (LAD) and that of the aortic root (Ao) were measured using a digital caliper with the corresponding inner edge‐to‐inner edge echocardiographic technique,^22^ and their ratio was calculated (LAD/Ao). At the level of the papillary muscles of the left ventricle at the midventricular level, the internal diameter of the right ventricle (RVID) and that of the left ventricle (LVID), and the thickness of the interventricular septum (IVS) and of the left ventricular posterior wall (LVPW) were measured using a caliper.

## 3 STATISTICS

The statistical analyses were carried out by a veterinarian with PhD‐level training in multivariate statistics (M.D.S.), using commercially available software (R Core Team 2020, version 4.0.2, R Foundation for Statistical Computing, Vienna, Austria; Excel: Microsoft Excel, Microsoft Office 2011, Microsoft Corporation, Bellevue, WA). The normality of data distribution was analysed using visual inspection of the plots and a Shapiro‐Wilk normality test. Any abnormal distribution of data was indicated.

**FIGURE 3:**
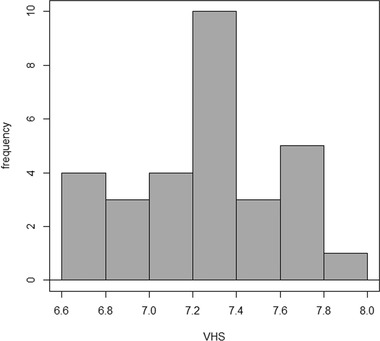
Histogram depicting the distribution of the VHS values measured on thoracic radiographs of 30 clinically normal pet guinea pigs

Prediction intervals were calculated following the American Society for Veterinary Clinical Pathology (ASVCP) guidelines for ≥ 20 and < 40 reference samples, for the radiographic study, or for 10 ≤ *x* < 20 samples, for the anatomical study.^23^ To illustrate the uncertainty of the small sample sizes, 90% confidence intervals (CI) were calculated, along with a histogram depicting data distribution (Figure 3), median, interquartile range (IQR), and minimum and maximum values for the radiographic study (Table 1), whereas median values and IQR are reported for the anatomical study (Table 2), as suggested by Friedrichs et al.^23^

**TABLE 1:** Radiographic study of 30 clinically healthy pet guinea pigs: Signalment and radiographic cardiac measurements obtained from right lateral thoracic radiographs

Variable	Median (IQR)	Range(min‐max)	Lower 90% C.I.	Upper 90% C.I.
Age* (years)	2.0 (1.0‐3.2)	0.6‐6.0	1.9	2.9
**BW (g)**	941 (800‐1047)	500‐1280	869	985
**LA (v)**	4.3 (4.1 −4.4)	3.7‐4.8	4.1	4.3
**SA (v)**	3.1 (3.0‐3.2)	2.8‐3.7	3.0	3.2
**LA (mm)**	26.7 (24.8‐27.9)	23.7‐31.7	26.0	27.3
**SA (mm)**	20 (19‐20.6)	17.3‐25.3	19.4	20.4
**LA + SA (mm)**	45.7 (44.1‐49)	41.4‐54.3	45.6	47.5
**VHS (v)**	7.4 (7.1 −7.6)	6.6‐8.0	7.2	7.4
CVC* (mm)	4.0 (3.7‐4.5)	3.0‐6.3	3.2	5.0
T5 length* (mm)	5.7 (5.3‐6.0)	4.7‐8.0	5.6	5.9

*Non‐normally distributed variables. BW, body weight; CVC, caudal vena cava maximum diameter; LA, cardiac long axis; SA, cardiac short axis; T5 length, cranio‐caudal length of the vertebral body of the fifth thoracic vertebra; v, number of vertebrae; VHS, vertebral heart score.

**TABLE 2:** Anatomical study of 10 guinea pigs: Signalment and cardiac anatomical measurements, and statistical comparison with previously published echocardiographic data^20^

Variable	Anatomically‐derived measurementsMedian (IQR)	Echocardiographic measurements^20^Median (IQR)	*P*‐value
**BW (g)**	539 (490‐563)	902 (822‐998)	<.001
**LA (mm)**	21.9 (21.2‐23)	NA	NA
**SA (mm)**	17 (15‐17.9)	NA	NA
**LA (v)**	4.5 (4.3‐4.5)	NA	NA
**SA (v)**	3.0 (3.0‐3.2)*	NA	NA
**VHS (v)**	7.5 (7.2‐8.0)	NA	NA
**LAD (mm)**	5 (4.5‐6.4)	6.9 (6.4‐7.6)	.02
**Ao (mm)**	2.6 (2.5‐2.8)	5.8 (5.3‐6.1)	<.001
**LAD/Ao**	1.6 (1.5‐1.9)	1.2 (1.1‐1.4)	.001
**LVID (mm)**	7 (5.9‐7.3)	s:6.8 (6.1‐7.4); d: 10.3 (9.3‐10.8)	.9; <.001
**RVID (mm)**	3.7 (3.5‐4.2)	d: 3.4 (2.8‐4.2)	.3
**IVS (mm)**	2.5 (2.4‐2.6)	s: 2.8 (2.6‐3.1); d: 2.1 (1.9‐2.3)	.02; .002
**LVPW (mm)**	2.8 (2.7‐2.9)	s: 2.9 (2.7‐3.2); d: 2.0 (1.8‐2.1)	.2; <.001

*Non‐normally distributed data (*P* = .03). Ao, aortic root diameter; BW, body weight; d, at end‐diastole; IVS, interventricular septum thickness; LA, cardiac long axis; LAD, left atrium diameter; LAD/Ao, ratio of the left atrium diameter to the aortic annulus diameter; LVID, left ventricular internal diameter; LVPW, left ventricular posterior wall thickness; RVID, right ventricular internal diameter; s, at end‐systole; SA, cardiac short axis; v, number of vertebrae; VHS, vertebral heart score.

With regard to the radiographic study, data comparison between sexes and between recumbencies (left versus right) was carried out by utilizing a Student's T‐test for independent groups. A Pearson's correlation or a Spearman's rank correlation test were used to assess whether a correlation existed between the VHS and age, the VHS and BW, LA and BW, SA and BW, LA+SA and BW, and T5 and CVC.

Echocardiographic measurements obtained from a population of 22 healthy pet guinea pigs utilized in a previously published study of the Authors’ research team^20^ were used to compare the present anatomical cardiac measurements with the echocardiographic findings (Table 2). A Mann‐Whitney test was carried out to compare the anatomically derived cardiac measurements with the echocardiographic variables,^20^ as well as to compare the anatomically derived VHS values with those obtained by radiography in the present study.

Interobserver reliability, with regard to the radiographic study, was assessed with the intraclass correlation coefficient (ICC).^24^, ^25^ The ICC estimates and their 95% CIs were calculated using Excel (Microsoft Excel; Microsoft Office 2011, Microsoft Corporation, Bellevue, WA) based on a mean‐rating (k = 3), absolute‐agreement, two‐way mixed‐effects model. Intraclass correlation coefficient reliability was rated as poor (ICC < 0.5), moderate (0.5‐0.75), good (0.76‐0.90), or excellent (> 0.90).^25^ A *P*‐value < .05 was considered significant.

## 4 RESULTS

### 4.1 Radiographic study

The conscious radiographic procedure was well tolerated by all the guinea pigs examined. No complications, such as respiratory distress, or injuries associated with physical restraint were observed.

On the right lateral recumbency radiographs, the heart extended from the caudal border of the second rib to the fourth intercostal space (IS) in five (16.7%) guinea pigs, from the second IS to the fourth IS in six guinea pigs (20%), from the second IS to the caudal border of the fifth rib in 10 guinea pigs (33.3%), and from the second IS to the fifth IS in nine guinea pigs (30%). The majority of the hearts (19/30; 63.3%) radiographically occupied 3‐3.5 ISs, while 11 of 30 (36.6%) occupied 2‐2.5 ISs. The VHS measurements were found to be normally distributed (*P* = .3), as depicted in the histogram (Figure 3).

Interobserver ICC reliability among the 3 raters of the VHS measurements in the guinea pigs was rated as moderate (ICC3k = 0.68; *P* = .0001). Intraclass correlation coefficient reliability was rated as moderate for T5 length (ICC3k = 0.56), CVC (ICC3k = 0.65), SA (v) (ICC3k = 0.70), and LA (v) (ICC3k = 0.71), whereas it was rated as good for SA (mm) (ICC3k = 0.78) and LA (mm) (ICC3k = 0.82).

**FIGURE 4:**
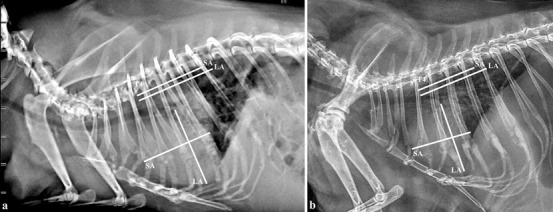
Right lateral thoracic radiographs of two guinea pigs (A, B) both diagnosed with dilated cardiomyopathy, showing the increase in the VHS measurements (60 kV, 200 mA, 20 mS, 4 mAs). LA, apicobasilar length of the heart; SA, width of the heart at its widest point perpendicular to LA; T4, fourth thoracic vertebra

A mean (±SD) VHS score of 7.3 ± 0.4 was found in the 30 presumed healthy guinea pigs. The VHS scores calculated in the two guinea pigs diagnosed with dilated cardiomyopathy were 8.9 v (SA: 4.2 v; LA: 4.7 v) (Figure 4A) and 8.4 v (SA: 4.0 v; LA: 4.4 v) (Figure 4B). Table 1 shows the statistics relating to all the signalment data and radiographic cardiac measurements taken from right lateral radiographs.

No differences emerged between the VHS measured in right (n = 10; 7.1 ± 0.4; 90%CI 6.8‐7.3) versus left (n = 10; 6.9 ± 0.4; 90%CI 6.6‐7.2) lateral recumbency (*P* = .41) or between male (7.2 ± 0.3; 90%CI 7.0‐7.3) and female (7.4 ± 0.4; 90%CI 7.3‐7.5) guinea pigs (*P* = .16). Body weight did not differ between male (992 ± 164.9 g) and female (871 ± 204 g) guinea pigs (*P *= .08). No correlation emerged between VHS and age (*P = *.53), or between VHS and BW (*P* = .26). Body weight was found to have an influence on the absolute cardiac SA (*P* = .02) and LA (*P* < .001) as well as on the sum of the SA and the LA (*P* < .001). Finally, the T5 and the CVC were not correlated (*P* = .3). The mean CVC/T5 ratio was 0.72 ± 0.10. Except for one guinea pig in which the CVC diameter was equal to the length of the T5, in the other 29 guinea pigs, the CVC was smaller than the T5.

### 4.2 Anatomical study

All hearts appeared macroscopically normal. The hearts of those guinea pigs positioned in right lateral recumbency extended from the second rib to the fifth intercostal space (n = 1), the fifth rib (n = 3), or the fourth intercostal space (n = 6). In left lateral recumbency, the heart had a more cranial topography, extending from the second to the fourth ribs (n = 7) or the fourth intercostal space (n = 3).

The VHS score obtained by anatomical dissection was not significantly different than that obtained by radiography (*P* = .3). The anatomically derived measurements obtained from 10 guinea pig hearts, along with the corresponding echocardiographic measurements obtained from a previous study carried out on guinea pigs,^20^ and the *P*‐values resulting from statistical comparison, are reported in Table 2.

## 5 DISCUSSION

The present study provides the first published report describing normal cardiac size assessment from thoracic radiographs using the VHS, together with an anatomical comparison, in presumed healthy pet guinea pigs. Species‐specific radiographic reference intervals of normality could prove useful in clinical practice in the objective evaluation of the cardiac silhouette of guinea pigs, which typically tend to present unspecific symptoms at the onset of the pathology.^5^

Radiographically, the majority of the hearts extended either to the fifth rib (30%) or to the fifth intercostal space (33.3%), therefore going beyond the caudal limit previously described in the literature (4th IS).^9^ Moreover, radiographically, only 20% (6/30) of the hearts occupied two ISs, as previously reported^9^; on the other hand, the majority (24/30; 80%) of the cardiac silhouettes occupied 2.5 to 3.5 ISs. The cranio‐caudal limits and number of ISs occupied by the guinea pig heart herein reported could prove useful for at‐a‐glance heart size assessment before obtaining more objective cardiac measurements using the VHS.^8^

No differences were observed between measurements taken in right versus left lateral recumbency. This finding is in line with studies carried out in chinchillas,^16^ agoutis,^26^ ferrets,^17^ laboratory mice,^18^ and dogs.^7^ However, it is in contrast with studies carried out on some dog breeds including beagles^11^, ^12^ in which the VHS was found to be significantly larger in right lateral recumbency, and with studies carried out on rabbits^15^ and whippets^10^ in which measurements were significantly larger in the left lateral view.

The results of the radiographic VHS obtained from right lateral projections in the guinea pigs in the present study (7.30 ± 0.4) were lower than those reported for other rodents, such as mice (9.1 ± 0.5),^18^ black‐rumped agoutis (8.0 ± 0.31; 7.68 ± 0.41),^26^, ^27^ and chinchillas (8.90 ± 0.72),^16^ but were also lower than the values reported for non‐rodents, such as African hedgehogs (8.16 ± 0.48),^19^ and dogs (9.7 ± 0.5).^7^ It, however, is fairly consistent with the ranges reported for rabbits (7.55 ± 0.38; 7.60 ± 0.39),^14^, ^15^ cats (7.5 ± 0.3),^13^ and black‐tailed prairie dogs (7.12 ± 0.42),^28^ and higher than the values reported for ferrets (5.25 ± 0.25; 5.57 ± 0.57).^17^, ^29^ This fact additionally validates the importance of obtaining species‐specific reference values of normality obtained under standardized conditions in order for the radiographic technique to accurately evaluate changes in the cardiac silhouette.

The radiographic VHS scores calculated in the two guinea pigs diagnosed with cardiac disease were, in both cases, above the upper reference limit of normality assessed in the presumed healthy guinea pigs. The cardiopathic guinea pigs included for comparison were chosen on the basis of the ultrasonographic evidence of an eccentric type of myocardial dilatation as concentric hypertrophy alone might not have determined a radiographically visible increase in the cardiac silhouette.^7^ Because of the small sample size as well as the absence of previously reported VHS values in guinea pigs with cardiac disease in the literature to compare our findings with, additional studies enrolling a larger number of guinea pigs with cardiac disease are necessary for determining the accuracy of the VHS in detecting cardiomegaly; however, this was beyond the scope of the present study.

It is important to note that normal VHS values do not necessarily rule out heart disease, as is the case with concentric hypertrophic dilatation, for instance.^7^ In chinchillas,^16^ ferrets,^29^ and cats,^30^ the VHS method proved to be scarcely sensitive, and only moderately accurate, in detecting selected cardiac abnormalities, such as cardiomegaly, or, in dogs, specific cardiac chamber enlargement.^31^

The CVC maximum diameter tended to be lower than the length of the vertebra dorsal to the tracheal bifurcation (T5) in the majority of the guinea pigs, confirming what had been observed in dogs.^7^ Moreover, the CVC/T5 ratio calculated in the present study was consistent with that reported for dogs.^7^ Therefore, it could be hypothesized that, as is the case with dogs, T5 could be taken as the upper normal limit for the CVC in healthy guinea pigs. The CVC/T5 ratio is a useful parameter in the assessment of the intravascular volume status of the patient; an increased ratio has been seen to be suggestive of right‐sided congestive heart failure in dogs.^32^

Interobserver variability was assessed to be moderate‐to‐good for all the radiographic indices in the present study. Potential sources of variation included the individual observers’ selection of cardiac and vertebral anatomic landmarks, and the transformation of the measurements of the cardiac long and short axes into units of vertebral length.^33^

From the anatomical study, it emerged that the cardiac cranial and caudal extension limits were, in the majority of cases (60%), consistent with those reported in the literature, that is from the second to the fourth IS, whereas, in four cases (40%), the heart had a more caudal extension than that previously described.^9^ The resulting median VHS score, assessed by anatomical dissection, was not statistically different from the corresponding median radiographic value, which pointed out the similarity between the anatomical and the radiographic techniques. On the other hand, the anatomical measurements of the aortic and left atrial internal diameters were significantly less than those of the echocardiographic measurements at end‐systole and end‐diastole reported in the literature.^20^ A possible explanation for the discrepancy was the post‐mortem change in the myocardium, leading to modifications of the chamber diameters,^34^ together with the loss of pressure and the consequent collapse of vessels such as the aorta.^35^ Another explanation might be the lower mean BW of the guinea pigs dissected. In fact, the above‐mentioned echocardiographic study showed a positive correlation between BW and the left atrial diameter.^20^ In addition, the LAD/Ao ratio calculated was almost double that assessed by echocardiography.^20^ This finding was probably ascribable to false left atrial dilatation secondary to postmortem myocardium deterioration. On the other hand, the internal diameters of the left and right ventricles were not different from the corresponding echocardiographic values measured at end‐systole and at end‐diastole, respectively.^20^ Analogously, the left ventricular posterior wall thickness value was not different from that reported by De Silva et al.^20^ at end‐systole. This could be ascribed to the postmortem myocardial contraction of the ventricles^34^;therefore, the measurements taken shortly post‐mortem are more likely comparable to the echocardiographic measurements taken at end‐systole rather than at end‐diastole.

A limitation of the present study was the small number of guinea pigs enrolled; a larger number (>120) would be necessary to establish normal reference intervals.^23^ Moreover, the radiographs in left lateral recumbency were obtained from ten guinea pigs only. The choice of using only manual restraint to obtain the radiographs was due to the fact that some anesthetic agents are known to alter cardiac measurements.^36^ The implementation of X‐ray positioning devices to decrease restraint stress, as well as the rapidity of the procedure and the absence of handling‐related complications, were at the base of such a decision. It is, however, advisable that guinea pigs with possible cardiorespiratory disease be sedated for radiography. Another limitation of the present study was the lack of additional diagnostic investigations, such as echocardiography, to ascertain the actual lack of cardiac disease in the guinea pigs studied as it has been demonstrated that guinea pigs with cardiac disease tend to be present few or unspecific symptoms.^5^, ^6^ Authors, indeed, cannot completely exclude the possibility that some of the clinically healthy animals could have had subclinical disease. However, based on the absence of clinical symptoms on physical examination and cardiac auscultation performed by a veterinarian with expertise in exotic small mammal medicine, as well as the absence of radiographic signs of cardiopulmonary disease in the totality of the animals included in the study, the guinea pigs were assumed by the authors to be likely free of cardiac disease. Therefore, also considering the radiographic differences in the cardiac silhouette seen between the presumed healthy guinea pigs and the two animals diagnosed with cardiac disease, the authors believe that the findings from this sample of clinically healthy guinea pigs can be valid for use as normal reference interval measurements. Additional studies involving healthy guinea pigs versus guinea pigs diagnosed with cardiopathy, are needed to strengthen the present findings and assess the accuracy of the VHS in detecting cardiomegaly in this species.

In conclusion, this study showed the feasibility of radiographic cardiac assessment in conscious guinea pigs and provided normal radiographic reference intervals which could prove useful for clinical use in the objective evaluation of cardiac size in guinea pigs.

## LIST OF AUTHOR CONTRIBUTIONS

### Category 1

(a) Conception and Design: De Silva
(b) Acquisition of Data: De Silva, Bo, Dora Genocchi, Grandis, Tagliavia, Roccaro
(c) Analysis and Interpretation of Data: De Silva, Bo, Dora Genocchi, Grandis, Baron Toaldo

### Category 2

(a) Drafting the Article: De Silva
(b) Revising the Article for Intellectual Content: Baron Toaldo, Grandis, Bo, Tagliavia, Roccaro, Dora Genocchi

### Category 3

(a) Final Approval of the Completed Article: De Silva, Bo, Dora Genocchi, Tagliavia, Roccaro, Grandis, Baron Toaldo

## CONFLICT OF INTEREST

The authors have declared no conflict of interest.
